# Boosting the cellulolytic oxidative and hydrolytic enzyme systems in *Myceliophthora thermophila* for the efficient enzymatic saccharification of lignocellulosic biomass

**DOI:** 10.1186/s40643-025-00967-5

**Published:** 2025-11-03

**Authors:** Rui Bai, Kun Yang, Yaru Wang, Yuan Wang, Xiaolu Wang, Tao Tu, Jie Zhang, Xiaoyun Su, Huoqing Huang, Bin Yao, Huiying Luo, Xing Qin

**Affiliations:** State Key Laboratory of Animal Nutrition and Feeding, Institute of Animal Science, Chinese Academy of Agricultural Sciences, Beijing, 100193 China

**Keywords:** Lytic polysaccharide monooxygenase, β-glucosidase, *Myceliophthora thermophila*, Enzymatic saccharification, Lignocellulosic biomass

## Abstract

**Abstract:**

The thermophilic fungus *Myceliophthora thermophila* serves as a vital platform for producing cellulolytic complex enzymes. However, their efficiency still requires enhancement to meet the cost-effective demands of lignocellulosic biomass conversion. Herein, secretome analysis revealed that the cellulolytic enzyme system of *M. thermophila* comprises the oxidative system consisting of lytic polysaccharide monooxygenases (LPMOs) and the hydrolytic system that includes endoglucanase, cellobiohydrolase, and β-glucosidase. Both in vitro supplementation and in vivo overexpression of *Mt*LPMOs with C1 or C1/C4 oxidizing activity enhanced the enzymatic saccharification of Avicel using *M. thermophila* fermentation broth, resulting in a maximum increase of 485% in oxidized cello-oligosaccharides production. Furthermore, the simultaneous enhancement of LPMO and β-glucosidase expression in *M. thermophila* significantly improved cellulose depolymerization and lignocellulosic biomass degradation. Total production of native and oxidized cello-oligosaccharides from pretreated corncob residue increased from 5.55 to 0.27 mg/mL in the wild-type strain to 8.72 mg/mL and 0.61 mg/mL in the engineered strain. Taken together, these findings highlight the synergistic interaction between the oxidative and hydrolytic enzyme systems for efficient saccharification of lignocellulosic biomass, providing valuable insights for enhancing the performance of commercial cellulolytic enzyme products.

**Graphical abstract:**

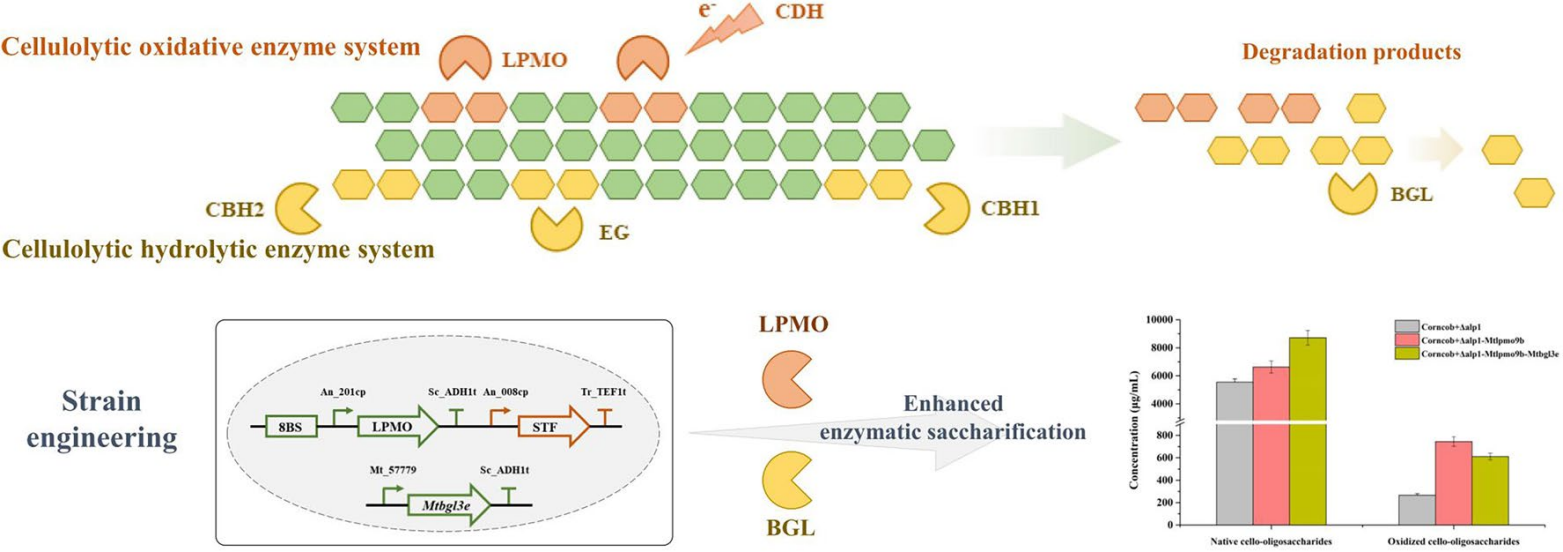

**Supplementary Information:**

The online version contains supplementary material available at 10.1186/s40643-025-00967-5.

## Introduction

Lignocellulosic biomass is the most abundant and renewable resource on the planet, with an estimated annual production of 180 billion tonnes worldwide (Deng et al. 2023). Due to its widespread availability, renewable nature, and minimal competition with food production, lignocellulosic biomass has attracted considerable attention as a promising feedstock for the production of value-added chemicals and fuels (Mujtaba et al. 2023). However, the chemical composition and complex physical structure create significant challenges for its breakdown and conversion into simple sugars suitable for biorefineries (Lorenci Woiciechowski et al. 2020; Zhao et al. 2012), resulting in increased production costs for bio-based products. Consequently, there is an urgent need to develop efficient strategies for the enzymatic saccharification of lignocellulosic biomass.

Cellulose, as the main structural component of lignocellulosic biomass, features a highly ordered crystalline structure that presents a formidable barrier to enzymatic degradation, posing challenges for its conversion into simple sugars (Houfani et al. 2020). Traditionally, the enzymatic degradation of cellulose was thought to rely on the coordinated action of key glycoside hydrolases, including cellobiohydrolase, endoglucanase, and β-glucosidase. However, these enzymes are often inefficient at breaking down the crystalline structure of cellulose. In recent years, several studies have uncovered the role of oxidoreductases in reducing cellulose crystallinity and enhancing cellulose accessibility to glycoside hydrolases (Barbosa et al. 2020; Guo et al. 2022; Long et al. 2022). Specifically, lytic polysaccharide monooxygenases (LPMOs), which are a type of oxidoreductase, catalyze the oxidative breakage of glycosidic linkages in the crystalline regions of cellulose, thereby creating new chain ends and making cellulose more susceptible to hydrolysis by glycoside hydrolases (Keller et al. 2020; Sørlie et al. 2023; Uchiyama et al. 2022). Based on this, the integration of LPMOs and glycoside hydrolases holds significant potential for improving the effectiveness of enzymatic degradation of cellulose.

To date, the interactions between individual LPMOs and glycoside hydrolases in vitro for cellulose depolymerization have been investigated. According to previous studies, the synergistic action of these enzymes depends on both the regioselectivity of the LPMO and the type of catalytic reaction performed by the glycoside hydrolase (Keller et al. 2020; Qin et al. 2022; Sørlie et al. 2023; Tokin et al. 2020; Uchiyama et al. 2022). For instance, C1/C4 oxidizing LPMOs could enhance the activity of both reducing and non-reducing end cellobiohydrolases, while C1 oxidizing LPMOs specifically promote the activity of non-reducing end cellobiohydrolases and inhibit that of reducing end cellobiohydrolases (Keller et al. 2020; Tokin et al. 2020). Additionally, both C1 and C1/C4 oxidizing LPMOs could boost the activity of endoglucanases, whereas C4 oxidizing LPMOs inhibit their activity (Keller et al. 2020; Qin et al. 2022). Nonetheless, several important issues remain to be addressed, particularly regarding the overall impact of LPMOs when combined with glycoside hydrolases in a complex host enzyme environment. For instance, multiple LPMOs, including C1, C4, and C1/C4 oxidizing LPMOs, are co-expressed with glycoside hydrolases in *Myceliophthora thermophila* for lignocellulosic biomass degradation (Qin et al. 2023b); Additionally, a deficiency of the glycoside hydrolase β-glucosidase has been observed in the industrial filamentous fungus *Trichoderma reesei* during the lignocellulosic biomass degradation process (Frassatto et al. 2021). Given the diverse types and varying expression levels of LPMOs and glycoside hydrolases found in lignocellulolytic microorganisms, the interactions among these enzymes in vivo for cellulose depolymerization are likely to be even more intricate. Therefore, an in-depth exploration of these interactions in a complex enzymatic environment is crucial for developing effective strategies for lignocellulosic biomass conversion.

*M. thermophila* is a thermophilic lignocellulosic biomass-degrading filamentous fungus known to produce a wide range of cellulolytic oxidoreductases and glycoside hydrolases (Berka et al. 2011). It has been widely used as a platform for the production of cellulolytic enzymes for the enzymatic saccharification of lignocellulosic biomass (Anu et al. 2022; da Rosa-Garzon et al. 2022; Singh 2016). Given the high cost of cellulolytic enzymes in lignocellulosic biomass biorefineries (Usmani et al. 2021), a comprehensive investigation and rational reformulation of the cellulolytic LPMOs and glycoside hydrolases produced by *M. thermophila* are necessary, which could significantly enhance the cellulose depolymerization efficiency and reduce the consumption of cellulolytic enzymes. While our previous studies have demonstrated the synergistic effects between individual LPMOs and glycoside hydrolases from *M. thermophila* for cellulose and lignocellulosic biomass degradation in vitro (Qin et al. 2022, 2023b), their synergistic interactions in vivo remain incompletely understood. In this study, the composition of oxidoreductases and glycoside hydrolases in *M. thermophila* under lignocellulosic biomass conditions was revealed by secretomic analysis. Subsequently, the biological functions of three major LPMOs with distinct regioselectivities in a complex enzyme environment for cellulose depolymerization were demonstrated both in vitro and in vivo. Furthermore, the cellulolytic oxidoreductase LPMOs and glycoside hydrolases in *M. thermophila* were upgraded to improve the efficiency of enzymatic saccharification of lignocellulosic biomass.

## Materials and methods

### Strains and plasmids

The major extracellular protease-deficient strain of *M. thermophila*, designated as Δ*alp*1, was created by deleting the gene MYCTH_2303011 from the original strain *M. thermophila* ATCC42464 (Li et al. 2023). The modified overexpression vector SESA containing a geneticin resistance cassette for homologous expression was chemically synthesized by Genewiz in Suzhou, China, according to the previous study (Rantasalo et al. 2018). The overexpression vector pPH1 containing a zeocin resistance cassette for homologous expression was constructed using the promoter of the histone H3-encoding gene (MYCTH_57779) from *M. thermophila* and the terminator of the alcohol dehydrogenase-encoding gene from *Saccharomyces cerevisiae*.

### Secretomic analysis of *M. thermophila* Δ*alp*1 cultured on corncob residue

*M. thermophila* Δ*alp*1 was cultivated on potato dextrose agar plates for 7 days to produce spores. The harvested spores were then concentrated to 10^6^/mL and used as an inoculum in liquid Vogel's medium supplemented with 2% corncob residue. The culture was incubated at 45 °C with shaking at 200 rpm for 3 days. Following this incubation, the extracellular proteins from the fermentation medium were collected and sent to Allwegene Technology (Beijing, China) for secretomic analysis. The data-independent acquisition mass spectrometry (DIA-MS) analysis was performed using a Thermo Scientific Vanquis Neo UHPLC system coupled with an Orbitrap Astral mass spectrometer. The parameters were configured as follows: a DIA m/z scan range of 380 to 980, isolation window widths of 2 Th, and a normalized AGC target set at 500%. The resulting data were analyzed using DIA-NN software (Demichev et al. 2020). The search parameters for DIA-NN included trypsin digestion with a maximum of one missed cleavage allowed; carbamidomethyl as a fixed modification; methionine oxidation and N-terminal acetylation as variable modifications; and a precursor false discovery rate set at 0.01.

### Homologous expression of major LPMOs with distinct regioselectivities

Three of the most abundant extracellular LPMOs with different regioselectivities (C1 oxidizing LPMO-*Mt*LPMO9B, C4 oxidizing LPMO *Mt*LPMO9J, and C1/C4 oxidizing LPMO-*Mt*LPMO9H) were selected for homologous expression in *M. thermophila* Δ*alp*1. The corresponding encoding genes, MYCTH_80312, MYCTH_79765, and MYCTH_46583, were cloned from the genomic DNA of *M. thermophila* Δ*alp*1 using gene-specific primers (Table S1). The resulting PCR products were assembled into the filamentous fungal expression vector SESA to create the overexpression vectors SESA-*Mtlpmo*9b, SESA-*Mtlpmo*9j, and SESA-*Mtlpmo*9h. Next, the targeted gene fragments from these overexpression vectors were amplified, precipitated, and transformed into *M. thermophila* Δ*alp*1 protoplasts using our previously established fungal transformation method (Li et al. 2023). Positive transformants were confirmed by analyzing the size of the PCR products with the primers YZ-SESA-F/R (Table S1).

Subsequently, a random selection of transformants was cultured on potato dextrose agar plates to produce spores for fermentation. The extracellular proteins harvested from the fermentation medium, comprising 75 g/L glucose, 10 g/L yeast extract, 0.15 g/L KH_2_PO_4_, 0.15 g/L K_2_HPO_4_, 0.1 g/L MgSO_4_·7H_2_O, 0.1 g/L CaCl_2_·2H_2_O, 0.1 mg/L biotin, and 1 mL/L trace element solution, were collected on the third day and subjected to SDS-PAGE analysis and purification. Following incubation with copper ions, the catalytic function of these recombinant LPMOs was verified by analyzing degradation products using Avicel as a substrate. The reaction mixture consisted of 4% Avicel, 1 μM LPMO, and 1 mM ascorbic acid in a pH 5.0 sodium acetate buffer. Control reactions were conducted without LPMO. The reaction was carried out at 45 °C with shaking at 1000 rpm for 24 h. The resulting products were analyzed by high-performance anion exchange chromatography coupled with pulsed amperometric detection (HPAEC-PAD) on a Dionex CarboPac PA1 column (Qin et al. 2023b). The elution program was as follows: a linear gradient from 0 to 10% B (1 M NaOAc in 0.1 M NaOH) over 10 min, followed by an increase from 10 to 30% B over 15 min, then a rapid rise from 30 to 100% B over 5 min, a subsequent decrease from 100 to 0% B over 5 min, and finally an isocratic elution with 100% A (0.1 M NaOH) for 5 min. The flow rate was maintained at 0.25 mL/min throughout the analysis.

### Functional characterization of LPMOs in complex enzyme environment for cellulose depolymerization in vitro and in vivo

For the in vitro experiments, cellulolytic enzymes were harvested from *M. thermophila* Δ*alp*1 cultured in liquid Vogel's medium supplemented with 2% corncob residue and 4 μM CuSO₄ for 3 days. The enzymatic reactions were carried out in a pH 5.0 sodium acetate buffer at 45 °C with shaking at 1000 rpm for 24 h. The reaction mixture consisted of 4% Avicel, 1 μM LPMO, and a four-fold dilution of the cellulolytic enzymes. Control reactions were performed by omitting either Avicel, LPMO, or the cellulolytic enzymes. For the in vivo experiments, cellulolytic enzymes were collected from *M. thermophila* Δ*alp*1, Δ*alp*1-*Mtlpmo*9b, Δ*alp*1-*Mtlpmo*9j, and Δ*alp*1-*Mtlpmo*9h, all cultured under the same conditions described above. The enzymatic reactions were conducted in a pH 5.0 sodium acetate buffer at 45 °C with shaking at 1000 rpm for 24 h. The reaction mixture included 4% Avicel and a four-fold dilution of the cellulolytic enzymes. Control reactions were performed by omitting either Avicel or the cellulolytic enzymes.

The resulting products were analyzed using HPAEC-PAD on a Dionex CarboPac PA1 column (Qin et al. 2023b). The elution program was modified as follows: a linear gradient from 0 to 10% B (1 M NaOAc in 0.1 M NaOH) over the first 25 min; a rapid increase from 10 to 100% B over the next 5 min; a return to 100% A (0.1 M NaOH) over 5 min; and finally, an isocratic hold at 100% A for an additional 5 min. The flow rate was maintained at 0.25 mL/min throughout the analysis.

### Optimization of the expression profile of cellulolytic oxidoreductases and glycoside hydrolases in *M. thermophila *Δ*alp*1

To construct an enhanced strain that co-expresses cellulolytic oxidoreductases and glycoside hydrolases, the β-glucosidase-encoding gene MYCTH_66804 was cloned from the genomic DNA of *M. thermophila* Δ*alp*1 using gene-specific primers pPH1_*Mtbgl*3e-F and pPH1_*Mtbgl*3e-R (Table S1). The resulting PCR product was then assembled into the filamentous fungal expression vector pPH1 to create the overexpression vector pPH1-*Mtbgl*3e. Subsequently, the targeted gene fragments derived from the overexpression vector were amplified, precipitated, and transformed into protoplasts of *M. thermophila* Δ*alp*1*-Mtlpmo*9b. Confirmation of positive transformants was achieved by analyzing the size of the PCR products using the corresponding primers YZ-pPH1-F/R (Table S1). Following this, a random selection of transformants was cultured on potato dextrose agar plates to produce spores for fermentation. The extracellular proteins harvested from the fermentation medium on the third day were subjected to SDS-PAGE analysis. The desired protein band was excised and analyzed using peptide mass fingerprinting with Thermo Scientific Q Exactive mass spectrometry, following a previously established method (Qin et al. 2022). This analysis employed trypsin digestion, carbamidomethylation, and methionine modification as parameters.

To characterize the changes in cellulolytic enzyme activities in the engineered strains *M. thermophila* Δ*alp*1-*Mtlpmo*9b and Δ*alp*1-*Mtlpmo*9b-*Mtbgl*3e, spores of these strains at a concentration of 10^6^/mL were used as an inoculum in Vogel's medium supplemented with 2% corncob residue and 4 μM copper sulphate. The culture was incubated at 45 °C and 200 rpm for 3 days to produce cellulolytic enzymes. The activities of cellulolytic enzymes, including endoglucanase activity, cellobiohydrolase activity, and β-glucosidase activity, were measured as follows: endoglucanase activity was assessed at 540 nm using 0.5% carboxymethyl cellulose sodium as the substrate, following the DNS reagent method (Qin et al. 2023a). Cellobiohydrolase and β-glucosidase activities were determined at 405 nm using 1 mM p-nitrophenyl β-D-cellobiose and 1 mM p-nitrophenyl β-D-glucopyranoside as substrates, according to a previously reported method (Qin et al. 2018).

### Functional characterization of engineered *M. thermophila* for lignocellulosic biomass degradation

The degradation ability of the engineered strains for cellulose depolymerization was evaluated through enzymatic saccharification on Avicel. The saccharification process was conducted in a pH 5.0 sodium acetate buffer at 45 °C with shaking at 1000 rpm for 24 h. The reaction mixture contained 4% Avicel and a four-fold dilution of the cellulolytic enzymes. Control reactions were performed without cellulolytic enzymes or Avicel. Meanwhile, pretreated corncob residue was utilized to assess the enzymatic saccharification potential of the engineered strains on lignocellulosic biomass. The corncob residue underwent pretreatment with 10% NaOH at a temperature of 80 °C for 4 h, using a solid-to-liquid ratio of 1:10 (Tsegaye et al. 2019). Enzymatic saccharification of the pretreated corncob residue was also carried out at 45 °C with shaking at 1000 rpm for 24 h. The reaction mixture included a pH 5.0 sodium acetate buffer, 4% pretreated corncob residue, and a four-fold dilution of the cellulolytic enzymes. Control reactions were conducted in the absence of either cellulolytic enzymes or pretreated corncob residue. The resulting products were then analyzed by HPAEC-PAD on a Dionex CarboPac PA1 column, following the previously described elution program.

## Results and discussion

### Secretome analysis revealed the cellulolytic oxidative and hydrolytic enzyme systems in *M. thermophila*

To better understand the cellulolytic oxidative and hydrolytic enzyme systems in *M. thermophila* under lignocellulosic biomass conditions, a secretome analysis of *M. thermophila* Δ*alp*1 cultured on corncob residue for 3 days was conducted. Based on the annotation of CAZymes from the genome (Berka et al. 2011; Grigoriev et al. 2013), 35 proteins from auxiliary activity (AA) families and 120 proteins from glycoside hydrolase families were identified among the extracellular proteins.

**Fig. 1:**
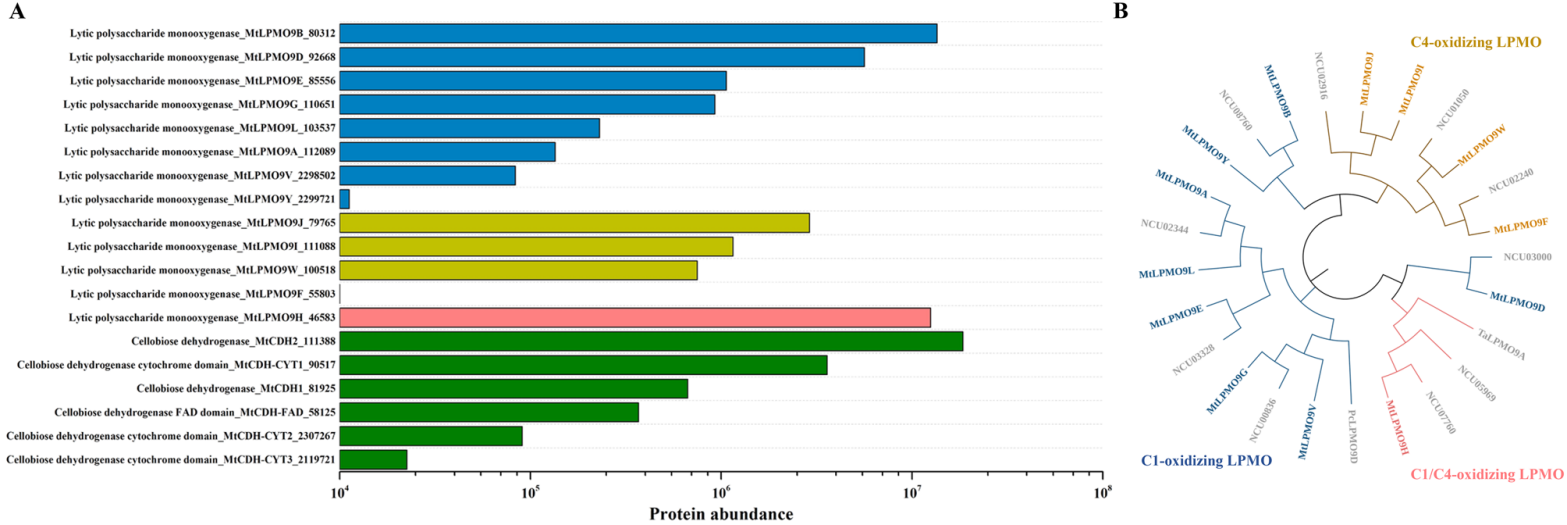
Protein abundance of cellulolytic oxidative enzymes in the secretome of *M. thermophila* Δ*alp*1 grown in liquid Vogel's medium supplemented with 2% corncob residue for 3 days (**A**). Evolutionary analysis of expressed AA9 LPMOs from *M. thermophila* Δ*alp*1. Colors indicated the oxidative regioselectivities: blue-C1 oxidizing LPMO, yellow-C4 oxidizing LPMO, and pink-C1/C4 oxidizing LPMO (**B**)

The cellulolytic oxidative enzyme system in *M. thermophila* consisted of LPMOs and cellobiose dehydrogenases (CDHs) from various AA families (Fig. 1A). Notably, thirteen cellulolytic LPMOs were highly expressed during the degradation of corncob residue, including *Mt*LPMO9A, *Mt*LPMO9B, *Mt*LPMO9D, *Mt*LPMO9E, *Mt*LPMO9G, *Mt*LPMO9H, *Mt*LPMO9I, *Mt*LPMO9J, *Mt*LPMO9L, *Mt*LPMO9V, and *Mt*LPMO9W from the AA9 family, as well as *Mt*LPMO16B and *Mt*LPMO16C from the AA16 family. Evolutionary analysis of the expressed AA9 LPMOs revealed that these enzymes exhibited different oxidative regioselectivities, including C1 oxidizing LPMO, C4 oxidizing LPMO, and C1/C4 oxidizing LPMO (Fig. 1B). This diversity suggested that there might exist combined effects among LPMOs for efficient cellulose depolymerization (Angeltveit et al. 2024; Sun et al. 2023). Additionally, six CDHs from the AA3 and AA8 families, including *Mt*CDH1, *Mt*CDH2, *Mt*CDH-FAD, *Mt*CDH-CYT1, *Mt*CDH-CYT2, and *Mt*CDH-CYT3, were observed to be expressed simultaneously, confirming the role of CDHs as natural electron donors in the catalytic cycle of LPMOs (Breslmayr et al. 2020; Felice et al. 2021; Hemsworth 2023).

**Fig. 2:**
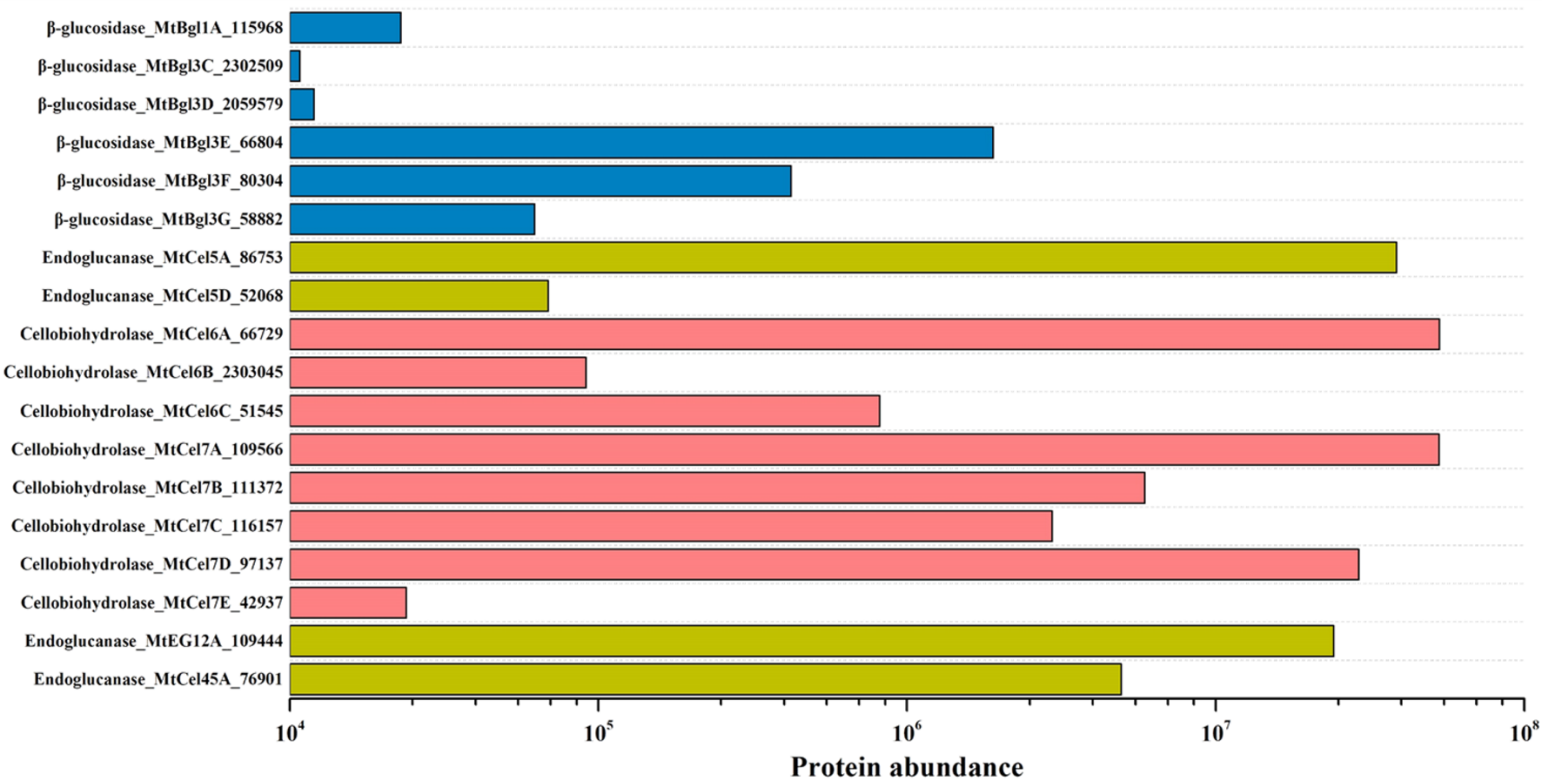
Protein abundance of cellulolytic hydrolytic enzymes in the secretome of *M. thermophila* Δ*alp*1 grown in liquid Vogel's medium supplemented with 2% corncob residue for 3 days

On the other hand, the cellulolytic hydrolytic enzyme system in *M. thermophila* comprised β-glucosidase, endoglucanase, and cellobiohydrolase from glycoside hydrolase families GH1, GH3, GH5, GH6, GH7, GH12, and GH45 (Fig. 2). Among these glycoside hydrolases, there were six β-glucosidases (*Mt*Bgl1A, *Mt*Bgl3C, *Mt*Bgl3D, *Mt*Bgl3E, *Mt*Bgl3F, and *Mt*Bgl3G), four endoglucanases (*Mt*Cel5A, *Mt*Cel5D, *Mt*EG12A, and *Mt*Cel45A), and eight cellobiohydrolases (*Mt*Cel6A, *Mt*Cel6B, *Mt*EG6C, *Mt*Cel7A, *Mt*Cel7B, *Mt*EG7C, *Mt*Cel7D, and *Mt*Cel7E). Significantly, the expression levels of β-glucosidases were found to be lower than those of endoglucanases and cellobiohydrolases by 1–3 orders of magnitude. This suggested that β-glucosidase might be a limiting factor in the cellulolytic hydrolytic enzyme system of *M. thermophila*, similar to the deficiency observed in other industrial filamentous fungi, such as *T. reesei* (Chan Ho Tong et al. 2024; Frassatto et al. 2021; Noguchi et al. 2021).

Based on the extracellular protein expression profile, it could be concluded that *M. thermophila* employs a combination of the LPMO-derived oxidative enzyme system and the hydrolytic enzyme system composed of endoglucanase, cellobiohydrolase, and β-glucosidase to efficiently depolymerize the cellulose component of lignocellulosic biomass. This finding suggests potential cooperative interaction mechanisms between the oxidative and hydrolytic enzyme systems during the cellulose depolymerization process. However, the interaction effects between LPMOs and glycoside hydrolases in the complex enzymatic environment associated with lignocellulose degradation remain to be elucidated, representing a significant scientific challenge that hampered a comprehensive understanding of efficient cellulose depolymerization mechanisms (Sørlie et al. 2023). While previous studies had analyzed the promoting and inhibiting effects of individual LPMOs and endoglucanases/cellobiohydrolases on cellulose depolymerization (Qin et al. 2022; Tokin et al. 2020; Uchiyama et al. 2022), the conclusions drawn from these studies might not provide sufficient guidance for the application of LPMOs, given the differences from the actual application scenarios. Therefore, an in-depth exploration of their interactions in a complex enzymatic environment was essential for developing effective strategies for lignocellulosic biomass conversion.

### LPMOs with distinct oxidative regioselectivities drove enhanced cellulose depolymerization in vitro and in vivo

**Fig. 3:**
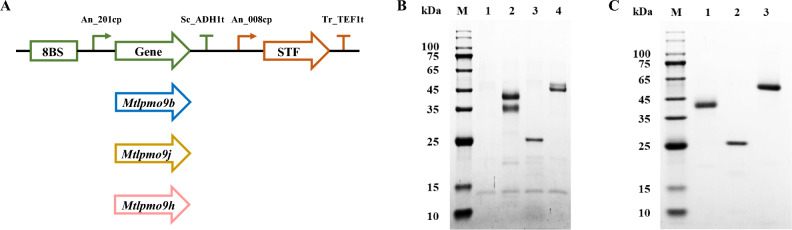
Schematic diagram of the homologous expression vectors SESA-*Mtlpmo*9b, SESA-*Mtlpmo*9j, and SESA-*Mtlpmo*9h (**A**). SDS-PAGE analysis of extracellular proteins produced by engineered strains grown in the fermentation medium for 3 days. 1, *M. thermophila* Δ*alp*1; 2, *M. thermophila* Δ*alp*1-*Mtlpmo*9b; 3, *M. thermophila* Δ*alp*1-*Mtlpmo*9j; and 4, *M. thermophila* Δ*alp*1-*Mtlpmo*9h (**B**). SDS-PAGE analysis of purified recombinant *Mt*LPMOs. 1, *Mt*LPMO9B; 2, *Mt*LPMO9J; and 3, *Mt*LPMO9H (**C**)

Considering that LPMOs were the dominant enzymes in the cellulolytic oxidative enzyme system, the most highly expressed LPMOs with different oxidative regioselectivities, *Mt*LPMO9B, *Mt*LPMO9J, and *Mt*LPMO9H, were selected for both ‌*in vitro* and in vivo experiments‌ to evaluate their interaction effects with glycoside hydrolases under complex enzymatic conditions. As shown in Fig. 3, *Mt*LPMO9B, *Mt*LPMO9J, and *Mt*LPMO9H were successfully expressed in the *M. thermophila* Δ*alp*1 strain, with distinct bands between 25 and 65 kDa observed in the SDS-PAGE analysis of extracellular proteins obtained from the engineered strains. Meanwhile, based on the analysis of degradation products from Avicel, purified recombinant *Mt*LPMO9B, *Mt*LPMO9J, and *Mt*LPMO9H exhibited C1 oxidizing, C4 oxidizing, and C1/C4 oxidizing activities, respectively (Fig. S1), consistent with the evolutionary analysis of the expressed AA9 LPMOs.

**Fig. 4:**
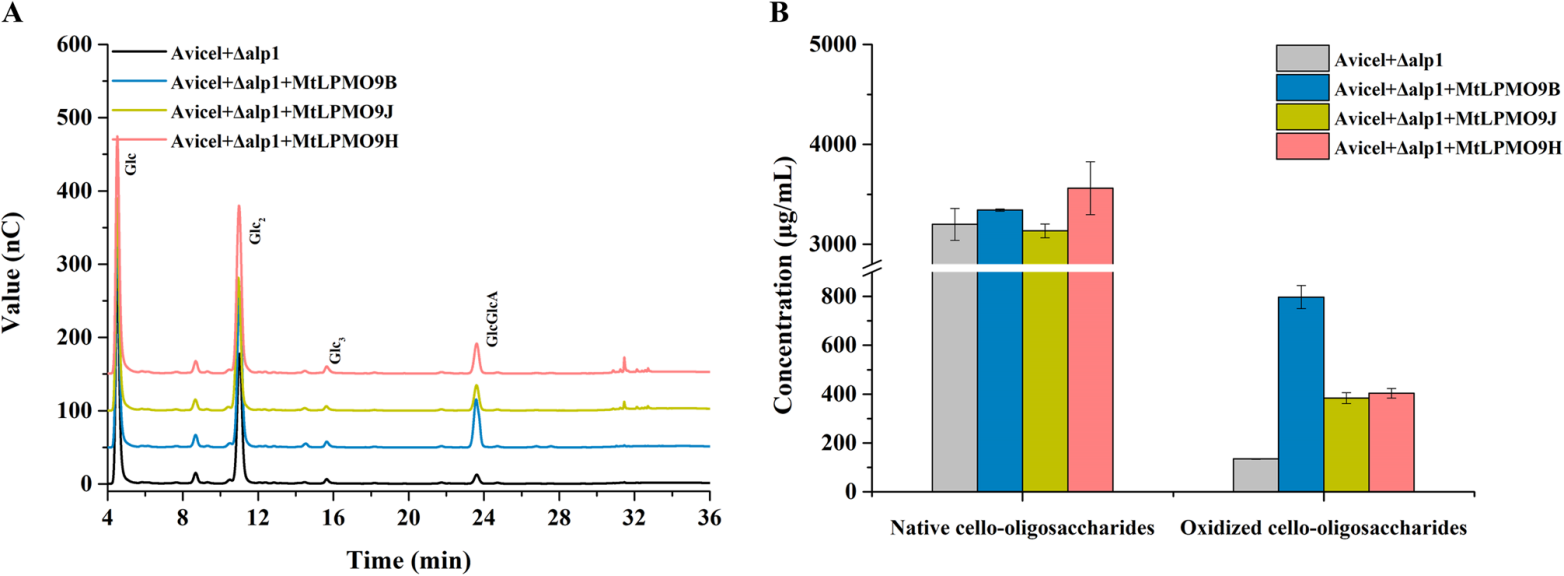
HPAEC-PAD analysis of the degradation products of Avicel by addition of 1 μM *Mt*LPMO to the fermentation broth of wild-type strain *M. thermophila* Δ*alp*1 in the pH 5.0 sodium acetate buffer at 45 °C with shaking at 1000 rpm for 24 h (**A**). The concentrations of degradation products of Avicel after 24 h, including both native and oxidized cello-oligosaccharides (**B**)

In the in vitro experiments, ‌purified recombinant *Mt*LPMOs were added to the fermentation broth of the wild-type strain *M. thermophila* Δ*alp*1 to evaluate changes in cellulose depolymerization capacity. According to the analysis of degradation products from Avicel, native cello-oligosaccharides, including glucose and cellobiose, as well as the oxidized cello-oligosaccharide cellobionic acid, were the primary degradation products during the cellulose depolymerization process (Fig. 4A). Compared to the control group without *Mt*LPMO, the groups supplemented with purified recombinant *Mt*LPMO9B, *Mt*LPMO9J, and *Mt*LPMO9H showed significant increases in the oxidized cello-oligosaccharides production, with improvements of 471%, 171%, and 186%, raising concentrations from 0.14 mg/mL to 0.80 mg/mL, 0.38 mg/mL, and 0.40 mg/mL, respectively (Fig. 4B). Besides, there was no significant improvement in the production of native cello-oligosaccharides except with the addition of *Mt*LPMO9H.

**Fig. 5:**
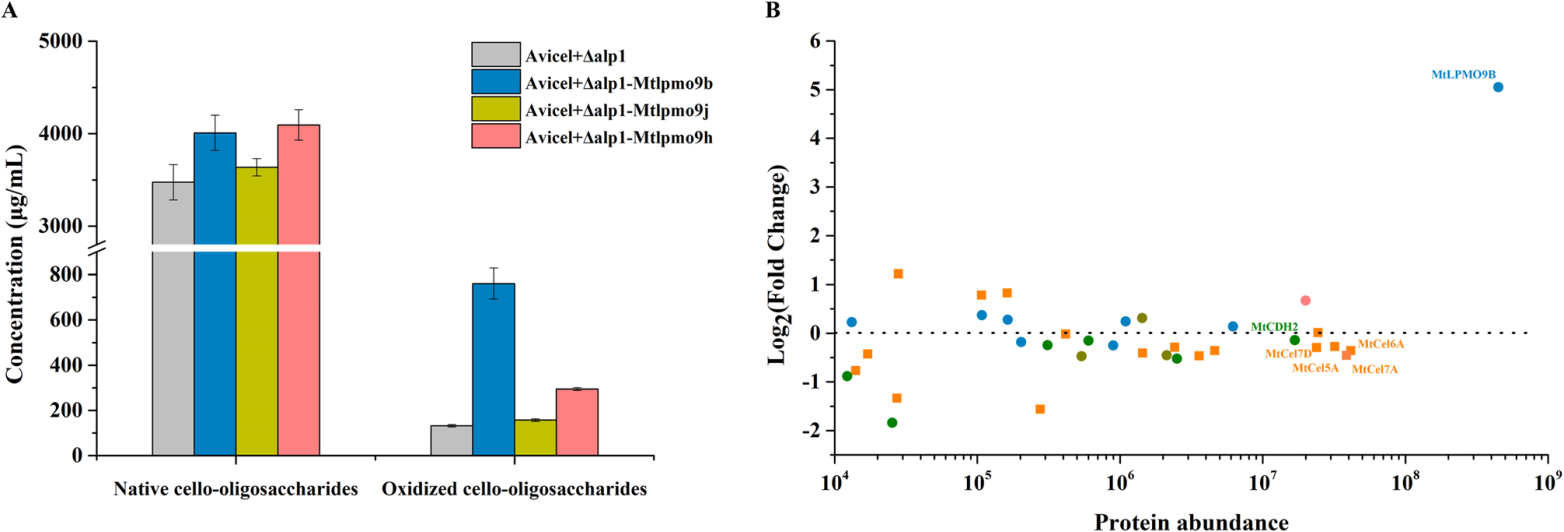
The concentrations of degradation products of Avicel by the fermentation broths of the engineered strains *M. thermophila* Δ*alp*1, Δ*alp*1-*Mtlpmo*9b, Δ*alp*1-*Mtlpmo*9j, and Δ*alp*1-*Mtlpmo*9h in the pH 5.0 sodium acetate buffer at 45 °C with shaking at 1000 rpm for 24 h (**A**). Protein abundance changes of cellulolytic oxidative and hydrolytic enzymes in the secretome of *M. thermophila* Δ*alp*1-*Mtlpmo*9b grown in liquid Vogel's medium supplemented with 2% corncob residue for 3 days. Shapes indicated the type of cellulolytic enzymes: circle-cellulolytic oxidative enzymes, square-cellulolytic hydrolytic enzymes (**B**)

In the in vivo experiments,‌ the fermentation broths of the engineered strains *M. thermophila* Δ*alp*1, Δ*alp*1-*Mtlpmo*9b, Δ*alp*1-*Mtlpmo*9j, and Δ*alp*1-*Mtlpmo*9h were directly used to assess changes in cellulose depolymerization capacity. Compared to the wild-type strain *M. thermophila* Δ*alp*1, the engineered strains *M. thermophila* Δ*alp*1*-Mtlpmo*9b and Δ*alp*1*-Mtlpmo*9h exhibited significant increases in the production of both native and oxidized cello-oligosaccharides. Specifically, there was a 15% increase in native cello-oligosaccharides production from 3.48 to 4.01 mg/mL and a 18% increase from 3.48 to 4.09 mg/mL for the engineered strains *M. thermophila* Δ*alp*1-*Mtlpmo*9b and Δ*alp*1-*Mtlpmo*9h, respectively (Fig. 5A). The improvement in native cello-oligosaccharides production could be attributed to the overexpression of *Mt*LPMOs, as there were no significant changes in the expression levels of highly expressed cellulolytic enzymes, including *Mt*Cel5A, *Mt*Cel6A, *Mt*Cel7A, and *Mt*Cel7D (Fig. 5B). On the other hand, the production of oxidized cello-oligosaccharides improved by 485% and 123%, increasing from 0.13 to 0.76 mg/mL and from 0.13 to 0.29 mg/mL for the same strains. In comparison, there was no significant increase in the production of native and oxidized cello-oligosaccharides using the fermentation broth of the engineered strain *M. thermophila* Δ*alp*1*-Mtlpmo*9j, which might be attributed to the low expression level of *Mt*LPMO9J in the liquid Vogel's medium supplemented with 2% corncob residue (Fig. S2).

**Fig. 6:**
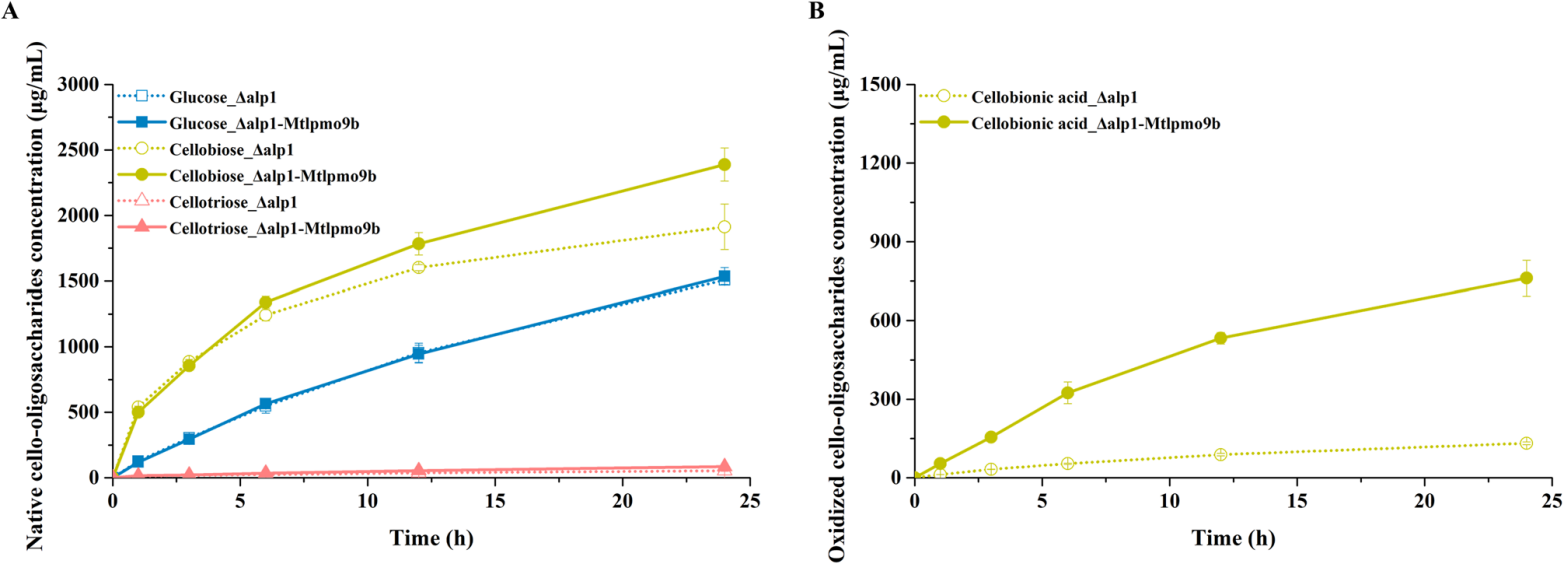
Time course analysis of the degradation products of Avicel by the fermentation broths of the engineered strains *M. thermophila* Δ*alp*1 and Δ*alp*1-*Mtlpmo*9b in the pH 5.0 sodium acetate buffer at 45 °C with shaking at 1000 rpm for 24 h, including native cello-oligosaccharides (**A**) and oxidized cello-oligosaccharides (**B**)

Based on our results regarding the interaction effects between LPMOs and glycoside hydrolases under complex enzymatic conditions, it was found that LPMOs with C1 or C1/C4 oxidizing activities significantly enhanced the cellulose depolymerization both in vitro and in vivo. However, this finding sharply contrasts with previous reports demonstrating that C1 oxidizing LPMOs inhibited the activity of reducing end cellobiohydrolases during cellulose depolymerization in simplified enzyme combination experiments (Keller et al. 2020, 2021; Sørlie et al. 2023). These contradictory results suggest that traditional single-enzyme models have inherent limitations and are insufficient for comprehensively deciphering the intricate relationship networks in native enzyme systems. Additionally, further time-course analysis of the degradation products from Avicel using fermentation broths of the engineered strains *M. thermophila* Δ*alp*1 and Δ*alp*1-*Mtlpmo*9b revealed a consistently high concentration of cellobiose throughout the cellulose depolymerization process (Fig. 6). This suggests that the activity of β-glucosidase in *M. thermophila* might be somewhat insufficient, which was consistent with the expression level profiles of cellulolytic hydrolytic enzymes in *M. thermophila*. Besides, it had been observed that the accumulation of cellobiose, an intermediate degradation product, could significantly feedback inhibit the hydrolytic activity of the cellulolytic enzymes such as endoglucanase and cellobiohydrolase, leading to reduced cellulose depolymerization efficiency (Angeltveit et al. 2024; Du et al. 2022; Zou et al. 2021). Therefore, simultaneously increasing the expression level of cellulolytic hydrolytic enzyme β-glucosidase would be crucial for further enhancing the cellulose depolymerization capacity of *M. thermophila*.

### Simultaneous enhancement of LPMO and β-glucosidase expression improved cellulose depolymerization and lignocellulosic biomass degradation

**Fig. 7:**
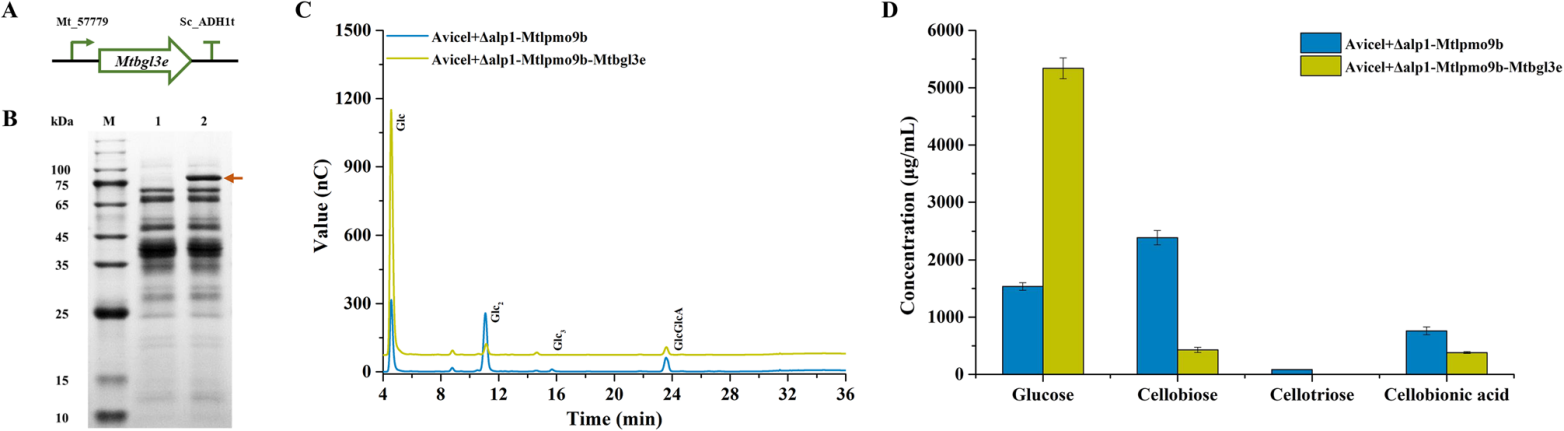
Schematic diagram of the homologous expression vectors pPH1-*Mtbgl*3e (**A**). SDS-PAGE analysis of extracellular proteins produced by engineered strains grown in the liquid Vogel's medium supplemented with 2% corncob residue for 3 days. 1, *M. thermophila* Δ*alp*1-*Mtlpmo*9b and 2, *M. thermophila* Δ*alp*1-*Mtlpmo*9b-*Mtbgl*3e (**B**). HPAEC-PAD analysis of the degradation products of Avicel by the fermentation broths of the engineered strains *M. thermophila* Δ*alp*1-*Mtlpmo*9b and Δ*alp*1-*Mtlpmo*9b- *Mtbgl*3e in the pH 5.0 sodium acetate buffer at 45 °C with shaking at 1000 rpm for 24 h (**C**). The concentrations of degradation products of Avicel after 24 h, including glucose, cellobiose, cellotriose, and cellobionic acid (**D**)

To further enhance the cellulose depolymerization capacity of *M. thermophila*, the most highly expressed extracellular glycoside hydrolase β-glucosidase, *Mt*Bgl3E, was selected for constitutive expression in the engineered strain *M. thermophila* Δ*alp*1-*Mtlpmo*9b using the overexpression vector pPH1. As shown in Fig. 7, SDS-PAGE analysis of extracellular proteins from the engineered strain revealed a distinct band at approximately 75 kDa. Subsequent analysis of this specific band through peptide mass fingerprinting confirmed the presence of peptides corresponding to *Mt*Bgl3E following trypsin digestion (Table S2), validating the successful overexpression of *Mt*Bgl3E in *M. thermophila* Δ*alp*1-*Mtlpmo*9b. Additionally, significant differences in β-glucosidase activity were observed between the engineered strains *M. thermophila* Δ*alp*1*-Mtlpmo*9b and Δ*alp*1*-Mtlpmo*9b*-Mtbgl*3e (Figure S3). In particular, the activity of β-glucosidase increased dramatically from 0.08 to 6.52 U/mL, representing an enhancement of two orders of magnitude.

In terms of the degradation capabilities of the engineered strains for cellulose depolymerization, *M. thermophila* Δ*alp*1-*Mtlpmo*9b*-Mtbgl*3e showed a significant improvement compared to the strain Δ*alp*1*-Mtlpmo*9b (Fig. 7). The total production of native cello-oligosaccharides increased by 44%, rising from 4.01 to 5.77 mg/mL following the constitutive expression of β-glucosidase in the engineered strain Δ*alp*1*-Mtlpmo*9b. Besides, glucose production saw a substantial increase, rising from 1.54 to 5.34 mg/mL. In contrast, the amount of cellobiose produced from the enzymatic degradation of Avicel decreased significantly from 2.39 to 0.43 mg/mL. These changes in the total production of native cello-oligosaccharides could be attributed to the enhanced production of β-glucosidase, which led to an increased rate of cellobiose hydrolysis. Furthermore, the inhibitory effects of the intermediate degradation product cellobiose on endoglucanase and cellobiohydrolase might be alleviated, thereby promoting cellulose depolymerization and increasing the production of native cello-oligosaccharides, especially glucose.

**Fig. 8:**
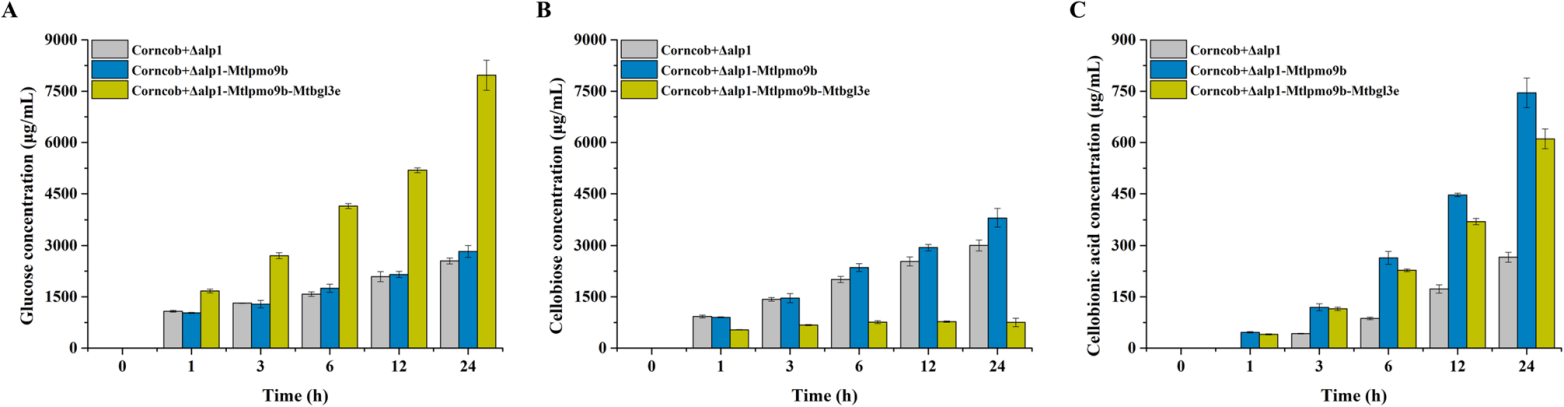
Time course analysis of the degradation products of pretreated corncob residue by the fermentation broths of the wild-type strain *M. thermophila* Δ*alp*1 and the engineered strains *M. thermophila* Δ*alp*1*-Mtlpmo*9b and Δ*alp*1*-Mtlpmo*9b*-Mtbgl*3e in the pH 5.0 sodium acetate buffer at 45 °C with shaking at 1000 rpm for 24 h, including glucose (**A**), cellobiose (**B**), and cellobionic acid (**C**)

Meanwhile, the extracellular proteins produced by these strains were utilized for the enzymatic saccharification of pretreated corncob residue to evaluate the effect of the engineered strains in degrading lignocellulosic biomass. As shown in Fig. 8, glucose, cellobiose, and cellobionic acid were the predominant degradation products during the degradation process of pretreated corncob residue. The total production of native and oxidized cello-oligosaccharides from the wild-type strain was 5.55 mg/mL and 0.27 mg/mL, respectively, after 24 h. In contrast, the engineered strains Δ*alp*1*-Mtlpmo*9b and Δ*alp*1*-Mtlpmo*9b*-Mtbgl*3e exhibited higher yields, with 6.63 mg/mL and 8.72 mg/mL of native oligosaccharides, and 0.75 mg/mL and 0.61 mg/mL of oxidized oligosaccharides, respectively. Significantly, glucose production showed a substantial increase from 2.55 mg/mL in the wild-type strain to 7.97 mg/mL in the engineered strain Δ*alp*1*-Mtlpmo*9b*-Mtbgl*3e, exhibiting a remarkable improvement of 213%. Collectively, the integrated engineering of cellulolytic oxidative and hydrolytic enzyme systems in *M. thermophila* enhanced cellulose depolymerization and lignocellulosic biomass degradation, leading to improved saccharification and higher oligosaccharide yields.

## Conclusions

In conclusion, secretome analysis revealed that both the LPMO-derived oxidative enzyme system and the hydrolytic enzyme system comprising endoglucanase, cellobiohydrolase, and β-glucosidase, play crucial roles in cellulose depolymerization by *M. thermophila*. Further enzymatic saccharification experiments showed that LPMOs with C1 or C1/C4 oxidizing activity could significantly enhance cellulose depolymerization both in vitro and in vivo. In addition, the simultaneous enhancement of LPMO and β-glucosidase expression in *M. thermophila* resulted in even greater improvements in cellulose depolymerization and lignocellulosic biomass degradation. Taken together, these findings highlight the synergistic interaction between cellulolytic oxidative and hydrolytic enzyme systems in lignocellulolytic microorganisms, providing valuable guidance for the updating and iteration of commercial cellulolytic enzyme products.

## Supplementary Information

Supplementary material 1.

## Data Availability

The datasets used and/or analysed during the current study are available from the corresponding author on reasonable request.

## Abbreviations

LPMOs: Lytic polysaccharide monooxygenases
DIA-MS: Data-independent acquisition mass spectrometry
HPAEC-PAD: High-performance anion exchange chromatography coupled with pulsed amperometric detection
CDHs: Cellobiose dehydrogenases
