# Supplementary material for: Boosting the cellulolytic oxidative and hydrolytic enzyme systems in Myceliophthora thermophila for the efficient enzymatic saccharification of lignocellulosic biomass
Source: Bioresour Bioprocess. 2025 Nov 3;12(1):126. doi: 10.1186/s40643-025-00967-5 (PMC12583286; doi:10.1186/s40643-025-00967-5)
Supplement: Supplementary file 1 — Supplementary material 1. [file 40643_2025_967_MOESM1_ESM.docx]

**Supporting Information**

**Boosting the** **cellulolytic oxidative and hydrolytic enzyme systems in *Myceliophthora thermophila* for the** **efficient enzymatic** **saccharification of lignocellulosic biomass**

Rui Bai, Kun Yang, Yaru Wang, Yuan Wang, Xiaolu Wang, Tao Tu, Jie Zhang, Xiaoyun Su, Huoqing Huang, Bin Yao, Huiying Luo*, Xing Qin*

State Key Laboratory of Animal Nutrition and Feeding, Institute of Animal Science, Chinese Academy of Agricultural Sciences, Beijing 100193, China

*Corresponding authors. E-mail address: luohuiying@caas.cn, qinxing@caas.cn, Tel.: +86-010-62599910.

**Table S1.** Primers used in this study.

| **Primer** | **Nucleotide sequence (5′→3′)*** |
| --- | --- |
| SESA_*Mtlpmo*9b-F | *TACTACAATTATTAATTAAA*ATGAAGTCCTTCACCCTCAC |
| SESA_*Mtlpmo*9b-R | *TAAGAAATTCGGATCCTCA*CTAGTGGTGGTGGTGGTGGTGGACGCACTGCGAGTAGTA |
| SESA_*Mtlpmo*9j-F | *TACTACAATTATTAATTAAA*ATGAAGCTCTCCCTCTT |
| SESA_*Mtlpmo*9j-R | *TAAGAAATTCGGATCCTCA*CTAGTGGTGGTGGTGGTGGTGGCAGGAGATGGGCGCGGGCCCA |
| SESA_*Mtlpmo*9h-F | *TACTACAATTATTAATTAAA*ATGTCCAAGGCCTCTGCTCTCCTC |
| SESA_*Mtlpmo*9h-R | *TAAGAAATTCGGATCCTCA*CTAGTGGTGGTGGTGGTGGTGCAAACACTGGGAGTACCACT |
| SESA-Vector-F | TGAGGATCCGAATTTCTTATGATTTATG |
| SESA-Vector-R | TTTAATTAATAATTGTAGTAGATTGG |
| YZ-SESA-F | TAAATGGGGTATATAAAGCACCC |
| YZ-SESA-R | TGAGAAAGCAACCTGACCTACAG |
| pPH1_*Mtbgl*3e-F | *ATCAACCAAGACATCATTCACA*ATGACCCTTCAAGCCTTTGC |
| pPH1_*Mtbgl*3e-R | *AAGAAATTCGGATCCTCA*TTAGTGGTGGTGGTGGTGGTGTACTCGGAAGCTCCCCGTCAGCC |
| pPH1-Vector-F | TGAGGATCCGAATTTCTTATGATTTATG |
| pPH1-Vector-R | TGTGAATGATGTCTTGGTTG |
| YZ- pPH1-F | GAAAACCCGCCCACTTAATCA |
| YZ- pPH1-R | TGAGAAAGCAACCTGACCTACAG |

^*^The homologous arm sequences are highlighted in italic.

**Table S2.** The peptide mass fingerprinting analysis of the recombinant *Mt*Bgl3E after trypsin digestion.

| **Protein name** | **Peptide** | **Unique** | **Start** | **End** |
| --- | --- | --- | --- | --- |
| *Mt*Bgl3E | R.GAAAWEAAHSSAAAALGK.L | Y | 30 | 47 |
|  | K.LSQQDKINIVTGVGWNK.G | Y | 48 | 64 |
|  | K.LSQQDK.I | Y | 48 | 53 |
|  | K.INIVTGVGWNK.G | Y | 54 | 64 |
|  | R.QRGEYMGAEFK.G | Y | 117 | 127 |
|  | R.QRGEYM(+15.99)GAEFK.G | Y | 117 | 127 |
|  | R.GEYMGAEFK.G | Y | 119 | 127 |
|  | R.GEYM(+15.99)GAEFK.G | Y | 119 | 127 |
|  | K.GC(+57.02)GIHVQLGPVAGPLGK.V | Y | 128 | 144 |
|  | K.HYILNEQELNR.E | Y | 184 | 194 |
|  | R.ETMSSNVDDR.T | Y | 195 | 204 |
|  | R.ETM(+15.99)SSNVDDR.T | Y | 195 | 204 |
|  | R.VLNVILK.Q | Y | 243 | 249 |
|  | K.SRLDDMVER.I | Y | 308 | 316 |
|  | K.SRLDDM(+15.99)VER.I | Y | 308 | 316 |
|  | R.LDDMVER.I | Y | 310 | 316 |
|  | R.LDDM(+15.99)VER.I | Y | 310 | 316 |
|  | R.DGIVLLK.N | Y | 353 | 359 |
|  | K.NDDGILPLK.K | Y | 360 | 368 |
|  | K.NDDGILPLKKPAK.L | Y | 360 | 372 |
|  | R.LNLDPWHNGNELVK.A | Y | 476 | 489 |
|  | K.LVYTIAK.R | Y | 550 | 556 |
|  | K.REQDYGTAVVR.G | Y | 557 | 567 |
|  | R.EQDYGTAVVR.G | Y | 558 | 567 |
|  | R.GDDTFPEGLFVDYR.H | Y | 568 | 581 |
|  | R.HFDKENIEPR.Y | Y | 582 | 591 |
|  | K.LAAGASGTATFSLR.R | Y | 680 | 693 |
|  | R.RDLSYWDTGR.G | Y | 695 | 704 |
|  | R.DLSYWDTGR.G | Y | 696 | 704 |
|  | R.GQWVVPEGEFGVSVGASSR.D | Y | 705 | 723 |

**Figure S1.** HPAEC-PAD analysis of degradation products of Avicel by 1 μM *Mt*LPMO in the pH 5.0 sodium acetate buffer containing 1 mM ascorbic acid at 45℃ with shaking at 1000 rpm for 24 hours.

**
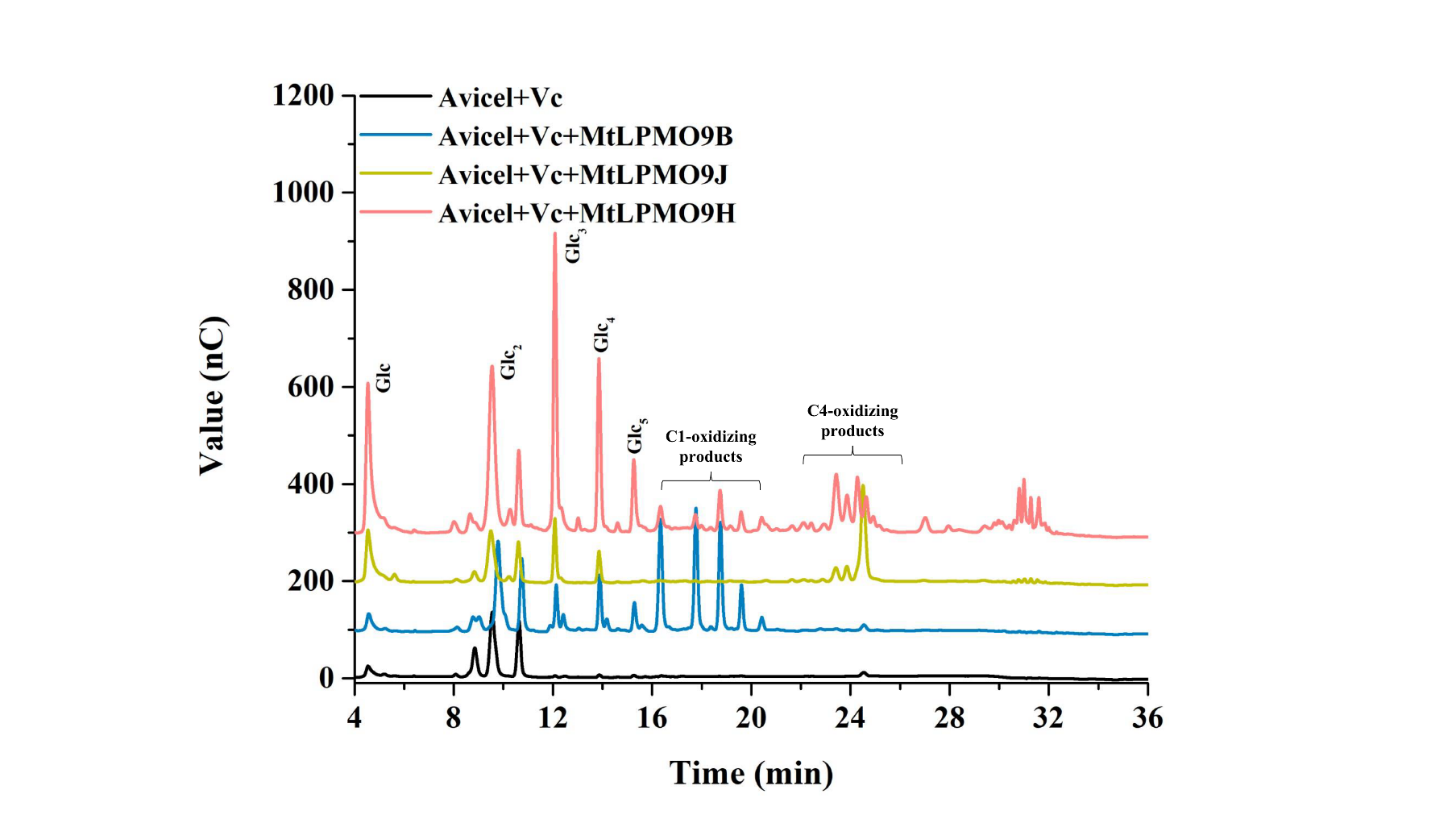
**

**Figure S2.** SDS-PAGE analysis of extracellular proteins produced by engineered strains grown in the liquid Vogel's medium supplemented with 2% corncob residue for 3 days. 1, *M. thermophila* Δ*alp*1; 2, *M. thermophila* Δ*alp*1-*Mtlpmo*9b; 3, *M. thermophila* Δ*alp*1-*Mtlpmo*9j; and 4, *M. thermophila* Δ*alp*1-*Mtlpmo*9h.

**
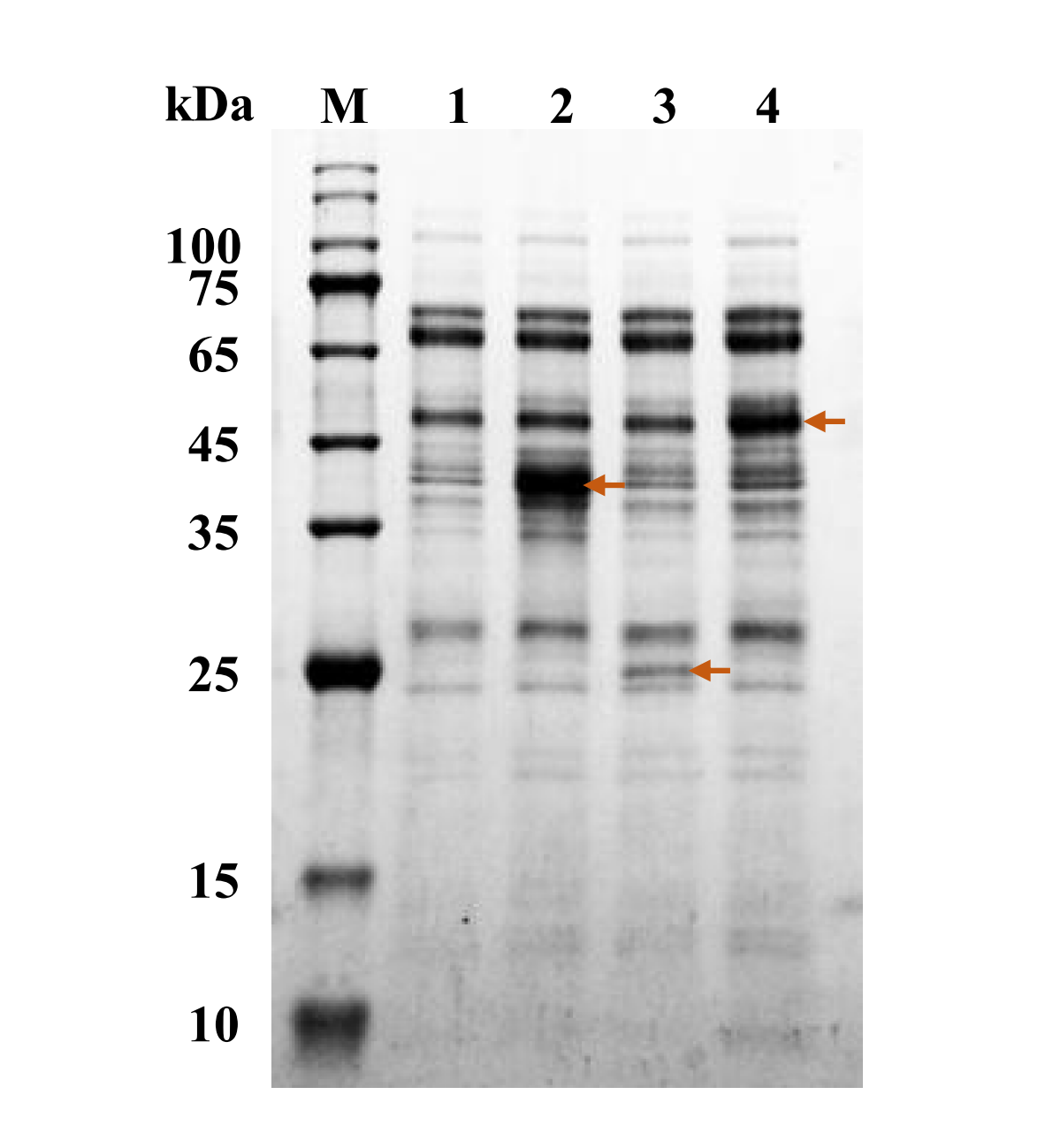
**

**Figure S3.** Extracellular activities of cellulolytic enzymes, including endoglucanase activity (A), cellobiohydrolase activity (B), and β-glucosidase activity (C), produced by the wild-type strain *M. thermophila* Δ*alp*1 and the engineered strains *M. thermophila* Δ*alp*1*-Mtlpmo*9b and Δ*alp*1*-Mtlpmo*9b*-Mtbgl*3e grown in the liquid Vogel's minimal medium supplemented with 2% corncob residue for 3 days.

**
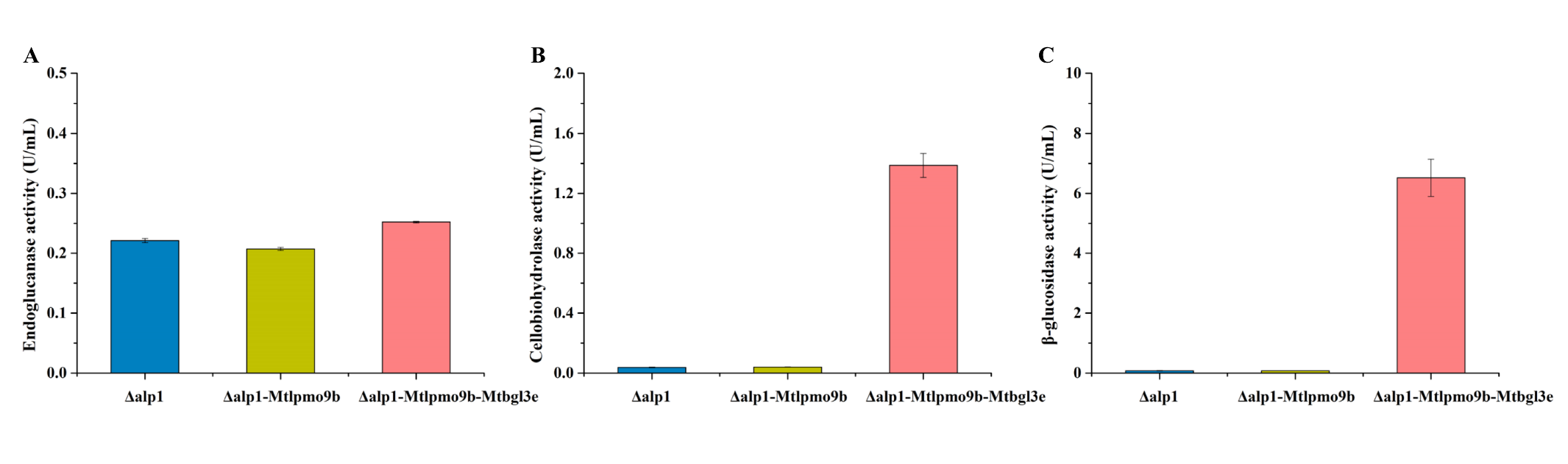
**
